# Challenges in anti‐aging medicine–trends in biomarker discovery and therapeutic interventions for a healthy lifespan

**DOI:** 10.1111/jcmm.17912

**Published:** 2023-08-23

**Authors:** Iuliana Popescu, Joris Deelen, Maddalena Illario, Jan Adams

**Affiliations:** ^1^ Barnstable Brown Diabetes Research Center University of Kentucky, College of Medicine Lexington Kentucky USA; ^2^ Max Planck Institute for Biology of Ageing Köln Germany; ^3^ Department of Public Health and EDAN Federico II University and Hospital Naples Italy; ^4^ Apollo Health Ventures Berlin Germany

**Keywords:** autophagy, biomarkers, centenarians, inflammaging, lifespan, lifestyles, rejuvenation, senescence, senolytics, senomorphics

## Abstract

We are facing a growing aging population, along with increasing pressure on health systems, caused by the impact of chronic co‐morbidities (i.e. cancer, cardiovascular and neurodegenerative diseases) and functional disabilities as people age. Relatively simple preventive lifestyle interventions, such as dietary restriction and physical exercise, are important contributors to active and healthy aging in the general population. However, as shown in model organisms or in 'in vitro' conditions, lifestyle‐independent interventions may have additional health benefits and can even be conceived as possible reversers of the aging process. Thus, pharmaceutical laboratories, research institutes, and universities are putting more and more effort into finding new molecular pathways and druggable targets to develop gerotherapeutics. One approach is to target the driving mechanisms of aging, some of which, like cellular senescence and impaired autophagy, we discussed in an update on the biology of aging at AgingFit 2023 in Lille, France. We underline the importance of carefully and extensively testing senotherapeutics, given the pleiotropism and heterogeneity of targeted senescent cells within different organs, at different time frames. Other druggable targets emerging from new putative mechanisms, like those based on transcriptome imbalance, nucleophagy, protein phosphatase depletion, glutamine metabolism, or seno‐antigenicity, have been evidenced by recent preclinical studies in classical models of aging but need to be validated in humans. Finally, we highlight several approaches in the discovery of biomarkers of healthy aging, as well as for the prediction of neurodegenerative diseases and the evaluation of rejuvenation strategies.

Aging poses one of the greatest challenges for modern medicine, as it is a major risk factor for chronic diseases such as cancer, cardiovascular and neurodegenerative diseases. As the global population continues to age, there is an urgent need to develop effective interventions and diagnostic tools to prevent illness and extend a healthy lifespan, thereby reducing the burden of age‐related diseases on healthcare systems.^1^ In the United States, it is estimated that healthcare spending for individuals over 65 years of age is three to five times higher than that for the younger population.^2^ Furthermore, the number of people over the age of 65 is expected to double by 2050 (to 2.1 billion), leading to a projected increase in healthcare costs of over 50%.^3^ In Europe, it is estimated that the annual cost of care for the population over 65 years of age will reach €1.5 trillion by 2050.^4^

Most basic research on aging is focused on identifying mechanisms contributing to this trait. Several hallmarks/pillars of aging have been defined and summarize the main processes underlying aging.^5^ Although these definitions are still suboptimal,^6^ they provide a nice framework used by many groups in the field to define which aspects of aging their research is focused on. Other scientists use more holistic approaches to study aging by, for example, investigating the interaction with the environment (mostly diet and exercise) or the loss/gain of function of certain genes contributing to multiple hallmarks at once. These studies have demonstrated that aging is a malleable process and that relatively simple lifestyle‐based interventions, such as dietary restriction, are likely a good starting point to improve healthy aging in the general population.^7^ However, research in model organisms has shown that targeting aging using lifestyle‐independent interventions may have additional health benefits.^8^ Therefore, many laboratories from universities and pharmaceutical companies are specifically focusing on identifying druggable targets and new molecular pathways for developing anti‐aging therapies.

One approach is to target the driving mechanisms of aging, like impaired autophagy and senescence. Inactivation of the mTOR pathway by interventions such as dietary restriction or with small molecule inhibitors like rapamycin (sirolimus), results in autophagy de‐repression and lifespan extension (in fruit flies, worms, and mice).^9^ Specific mTORC1 inhibitors (rapamycin analogs/‘rapalogs’, i.e. temsirolimus, tacrolimus, everolimus, etc) were developed as immunosuppressants and anticancer drugs, although they have proven to only be effective in few types of cancers. However, extensive data suggest that rapamycin and rapalogs may have positive effects on age‐related conditions and possibly on human longevity.^10^ For instance, the randomized, double‐blind, placebo‐controlled trial PEARL (NCT04488601/www.ClinicalTrials.gov) was recently initiated in the U.S. to assess the long‐term efficacy of rapamycin in reducing clinical outcomes associated with declining health and aging in healthy old adults. Biotech companies like Calico and Samsara Therapeutics are developing lead molecules that target the lysosome, an important cellular component of the autophagy cycle, with potential utilization in neurodegenerative diseases (www.calicolabs.com; www.samsaratherapeutics.com/our‐science/the‐lysoseeker‐platform).

Other therapeutic options appear as new molecular targets involved in the autophagic process are discovered. It is the case of the cardiac acyl‐CoA binding protein (ACBP), an extracellular inhibitor of autophagy, and subsequently, a promotor of heart aging in humans. Its depletion with monoclonal anti‐ACBP antibodies in mice decelerated the degradation of cardiomyocyte function during aging^11^, which indicates a new gero‐druggable pathway.

Cellular senescence is another potentially druggable mechanism that has been much ‘in focus’ to try to prevent or treat many age‐associated pathologies, including cardio/cerebrovascular, neurodegenerative, metabolic, and malignant diseases. Accumulated senescent cells (SenC) have deleterious effects due to the induced proinflammatory microenvironment that supports chronic low‐grade inflammation (‘*inflammaging*’) and possible tumor development, and accelerate other aging mechanisms which will progress concomitantly (‘*the geroscience hypothesis*’).^12^ In 2011, Baker et al. reported for the first time that inducible clearance of p16^Ink4a^ expressing SenC in aged mouse tissues is beneficial in delaying several aging–associated phenotypes.^13^ The depletion of SenC in aged organisms, either via genetic ablation or pharmacologically with senolytics, may have therapeutic benefits by alleviating a series of age‐associated comorbidities and thus improving healthspan and even lifespan, as shown in lower organisms. However, recent studies have shown the presence of highly senescent cells (p16^+^; p21^+^) in many young tissues where they may fulfill regeneration tasks and therefore, their ablation can trigger tissue damage in a young organism. For instance, ablation of ‘*sentinel*’ p16^+^ mesenchymal cells in the lungs of young mice leads to impaired restoration of the airway barrier upon injury^14^ and the removal of the highly senescent liver sinusoids within the middle‐aged hepatocytes, triggers collagen deposition and liver fibrosis in experimental animals.^15^ This positive face of senescence is most likely an extension of its primordial beneficial functions in embryo development, tissue regeneration or wound healing, or limitation of tumor development in young organisms.^16^ Altogether, this points out that the development of senolytics (and other senescence‐related interventions) has to be carefully conducted and tested in validated preclinical models and then in large, randomized clinical trials to prove their safety and benefits, including the pace and routes of administration. The most tested senolytics in clinical trials are either repurposed drugs, like dasatinib (an inhibitor of tyrosine kinase receptor used in the treatment of certain forms of leukemia), or plant flavonoids, such as quercetin and fisetin, alone or in combination with dasatinib (they interfere with the PI3K/Akt/mTORC1 pathway), which have previously shown a good safety profile (see the review of Chaib et al. for an update on senolytics in clinical trials).^12^ Newer classes of senolytics target the anti‐apoptotic proteins of the BCL‐2 family (i.e. navitoclax) and proteasomal degradation of this pro‐apoptopic family (the ‘spymicins’). A second generation has resulted from High Throughput Screening (HTS) and includes for instance galactose‐modified prodrugs and nanoparticles targeting the lysosomes.^17^

Also, some repurposed drug molecules have demonstrated new seno‐modulator characteristics, besides their classical mechanism of action. For example, the psychostimulant and anti‐depressant methylphenidate (MPH); its primary action is to activate dopamine release, but it is also able to activate the protein phosphatase 2A (PP2A), a neuronal protein whose activity decreases in the aging brain of zebrafish and mice.^18^ Activated PP2A has anti‐senescent properties in the neurons of these two species but might be also related to the fact that polymorphisms of the PP2A gene predispose human subjects to mental illness and cognitive impairment^19^ that can be attenuated by MPH treatment at advanced ages.^20^ From this point of view, PP2A remains a promising gero‐target but the real effects of its activation in preventing neuronal aging in humans are yet to be better documented.

Targeting particular seno‐antigens at the surface of SenC opens the possibility of developing senolytic vaccines with systemic effects in aged organisms. We hereby mention the glycoprotein nonmetastatic melanoma protein B (GPNMB). This seno‐antigen is critical for the survival of senescent human vascular endothelial cells,^21^ highly present in pathologies associated with vascular dysfunction, like atherosclerosis. This finding opened the possibility of targeting GPNMB–positive endothelial SenC to eliminate them, similar to senolytic therapy. Suda et al. provided evidence that this approach was indeed feasible when they immunized mice against GPNMB and found an improvement of vascular function in models of atherosclerosis, together with lifespan extension in progeroid mice.^22^

However, we should not forget about the other arm of senotherapy – the senomorphics, molecules that inhibit the production and secretion of SASP (senescent‐associated secretory phenotype) by SenC. Unlike the senolytics, the senomorphics need to be continuously administered, which might induce more side effects. On the other hand, targeting only SenC displaying SASP can be more finely adjustable than targeting the entire SenC population, which may also include beneficial SenC. Actual clinical trials are oriented towards testing small molecules which interfere with the main transcriptional regulators/pathways of SASP (e.g. mTOR, TNFα, NF‐κB or JAK/STAT inflammatory pathways),^23^ which also make them useful in the treatment of aging‐associated pathologies like cancers and chronic inflammatory diseases (Figure 1).

**FIGURE 1:**
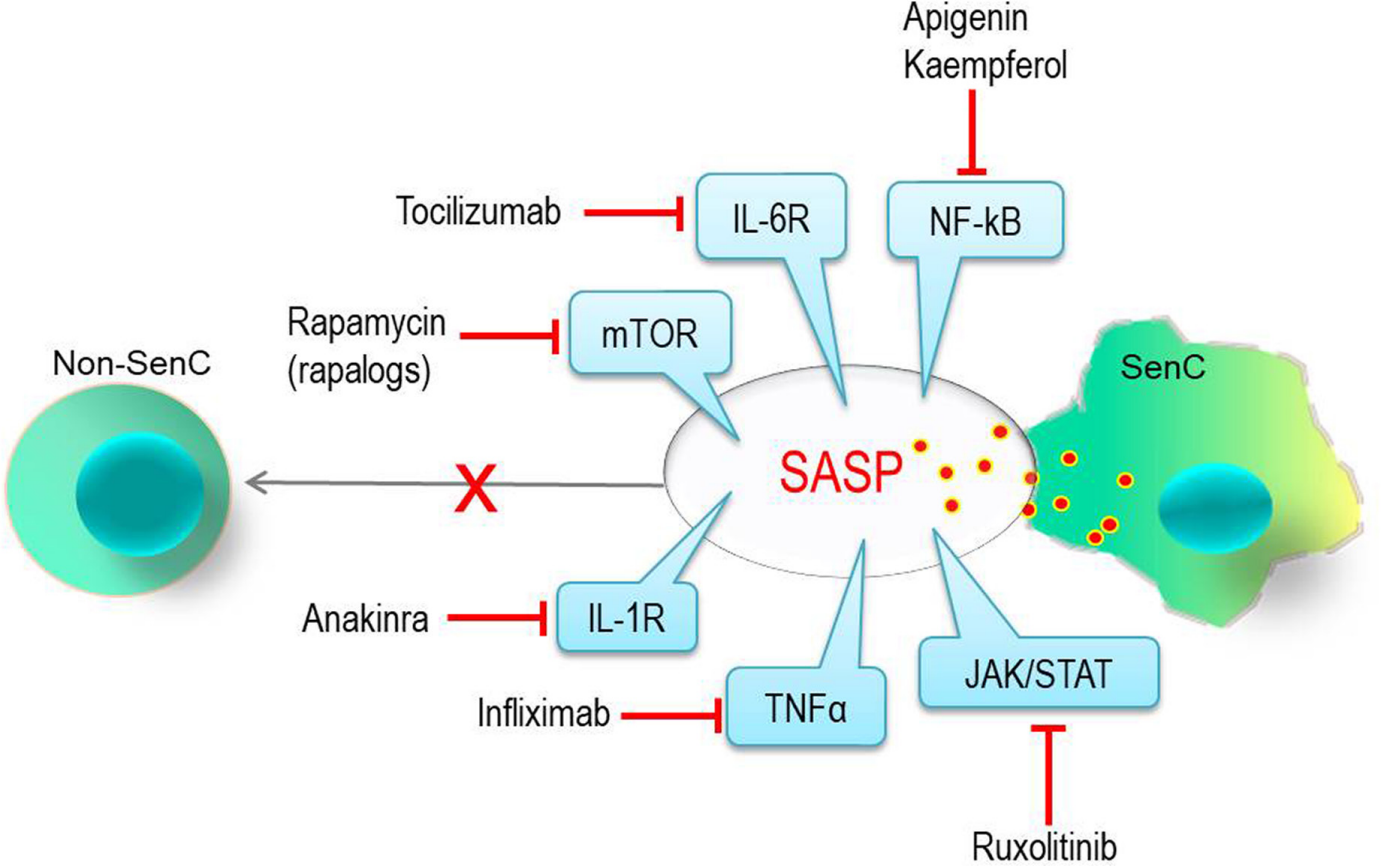
Senomorphic molecules (and their targets) tested in clinical trials in old adults (>65 years) for: breast cancer, colorectal cancer, Covid‐19, etc. (apigenin, kaempferol); age‐related sarcopenia, heart failure, transplantation, solid tumors, longevity, etc. (rapamycin); rheumatoid arthritis, various types of cancers, Covid‐19, etc. (tocilizumab); cytokine storm (in Covid‐19), lymphoma, gout, CKD, heart failure, T2D, pancreatic cancer, etc. (anakinra); glaucoma, inflammatory bowel disease, psoriasis, rheumatoid arthritis, etc. (infliximab); prostate cancer, HCC, leukemia, etc. (ruxolitinib). www.ClinicalTrials.gov. SASP, senescent‐associated secretory phenotype (comprising pro‐inflammatory cytokines, chemokines, growth factors, and extracellular matrix proteases); SenC, senescent cell.

Because senescent cells rely mainly on the glutamine catabolism for survival and the biosynthesis of SASP^24^, targeting the key enzyme GLS1 glutaminase with small inhibitory molecules (i.e. CB‐839, SP600125) could also restore age‐associated phenotypes, as demonstrated by Choudhury et al. in a progeroid mouse model.^25^ Another way to interfere with the SASP biosynthesis emerged from observations in patients infected with HIV under long active antiretroviral therapy, including certain protease inhibitors like atazanavir; such molecules (which target the ZMPSTE24 mammalian protease) cause accelerated premature senescence in these patients but treatment cessation seems to revert the senescent features and SASP production, as demonstrated in animal models.^26^

Nevertheless, findings from evidence‐based medicine have changed some paradigms around the beneficial effect of SASP inhibition. For a long‐time, aspirin was suggested to be a potential senomorphic drug, due to its anti‐oxidant and anti‐inflammatory properties. However, recent data from the ASPREE large clinical trial found that daily low‐dose aspirin in healthy older adults without previous cardiovascular events does not prolong healthy and independent living, but surprisingly, might predispose to higher mortality by cancer.^27^ Based on this finding, the guidelines of the American Heart Association recommend against the routine use of low‐dose aspirin in this category of old adults.^28^

Overall, a more detailed characterization of each SAPS phenotype and SenC subpopulation which produces it seems to be pivotal in initiating personalized anti‐aging approaches. In addition, a still unsolved problem is how to specifically quantify senescence and senolytic/senomorphic effect in individual organs in order to establish the right intervention. Lastly, SenC could, at least hypothetically, be reprogrammed with senoreverter molecules to re‐enter the cell cycle and ‘rejuvenate’ tissues via the iPSCs obtained from senescent and centenarian cells.^23^ In fact, the iPSCs (inducible pluripotent stem cells) approach is based on the results obtained first in adult mouse and human fibroblasts that have been reverted in vitro to an undifferentiated, highly proliferative stem cell state (iPSCs) by the forced expression of four critical transcription factors – Oct4, Sox2, Klf4 and c‐Myc, called Yamanaka reprogramming factors.^29^ Then, this in vitro approach was translated in vivo by several laboratories. For instance, researchers used injected adenoviruses to express the Yamanaka reprogramming factors in a progeria and glaucoma murine model and reversed the epigenetic age, additionally to retina regeneration.^30^ However, while very efficient in vitro to reverse the biological age to that of the embryo, the full reprogramming to iPSCs can be tumorigenic in vivo. This is why partial reprogramming is considered more appropriate to avoid tumorigenesis while preserving cellular identity.

Chemical rejuvenation with reversal of transcriptomic age independent of iPSCs induction was also described.^31^

Interesting findings of several recent pre‐clinical studies suggest new putative pathways and molecular targets for the development of geroprotectors. However, we have to keep in mind that most of these findings were obtained in the classical animal models of aging, so more studies are needed in human tissues and cells to determine their translatability.

A recent elegant study identified the nuclear RNA‐Pol II as a possible therapeutic target, starting from the interesting observation that the transcriptional elongation speed of the enzyme is increased with aging across multiple species, including *Caenorhabditis elegans*, *Drosophila* *melanogaster*, mice and rats, and certain human cell lines.^32^ As a consequence, genotoxic stress consisting of transcriptional errors, modifications in splicing, and the appearance of inefficient RNA forms, might occur and impact the function and lifespan of affected cells.^33^ Interestingly, the expression‐enrichment analysis showed that the genes with a high Pol II speed are those preponderantly involved in metabolic and catabolic processes, but there are many differences between their expressions in humans versus other species. Therefore, several strategies for slowing down the elongation speed of the enzyme and increasing the lifespan (demonstrated in worms and fruit flies) were finally proposed by Debes et al.: dietary restriction and lowering the signaling of the insulin‐IGF1 somatotropic axis (two lifespan‐extending interventions), genetic modification of the speedy Pol II by gene‐editing (with CRISPR‐Cas9) or, another demonstrated possibility, the overexpression of certain histones (e.g H3 and H4) to increase the nucleosomal density.^32^

Mammalian nesprins are another example of nuclear targets for geroprotection. These proteins anchored in the nuclear envelope of cells promote autophagic recycling of the nuclear components (“nucleophagy”) and control nuclear architecture and nucleolar size,^34^ a determinant of longevity.^35^ An active, enhanced nucleophagy delays aging in mice and nematodes while a deficient one, due to nesprin‐2 (the ANC‐1 ortholog in *C*. *elegans*) impairment for instance, predisposes to tumorigenesis in germline organs, in the same species^36^; additionally, nesprin‐2 polymorphisms were associated with ovarian cancer and endometrioses in women.^37^ Thus, strategies to stabilize nesprins (that can be perturbed by stress) or enhance their activity could at least hypothetically, promote rejuvenation and a healthy span of tissues like gonads and ovaries.

And also related to ovarian aging, we should mention another promising target, the bridge integrator 2 (BIN2) protein, a regulator of the mTOR pathways, enriched in ovaries and oocytes. Deletion of BIN2 in ovaries improved ovarian function and fertility in aged mice,^38^ most likely via mechanisms of phosphorylation, resulting in the derepression of nicotinamide nucleotide transhydrogenase, a ROS scavenger. Inhibition of BIN2 phosphorylation with a BIN2‐penetrating peptide preserved the ovarian function in aging mice or after chemotherapy.^38^

Studies performed in the vascular endothelium suggested that deterioration of vascular function is the driving factor of organismal aging^39^ because of the positive role played by VEGF signaling in most organs and tissues.^40^ Experiments of loss of function or gain of function of VEGF in aged mice demonstrated that optimal VEGF signaling is necessary to counteract age‐related multi‐organ chronic inflammation and maintain healthy (and non‐tumoral) aging phenotypes in many tissues (adipose, liver, muscle, bone), resulting in the extension of lifespan.^39^

The Imidazoline type 1 receptor (I1R) is another potential surface molecular target that can be exploited in pharmacological geroprotection at advanced ages. This was suggested after it was determined by HTS that its agonist, the antihypertensive drug rilmenidine, induces a transcriptional profile similar to caloric restriction in *C*. *elegans* and in the liver and kidney of mice.^41^ However, it is not yet clear if the longevity benefit of rilmenidine in these animal models is entirely dependent on the imidazoline receptor activity or if other independent mechanisms, like activation of key autophagy genes, are involved.

A summary of these putative molecular targets for the development of geroprotectors is presented in Table 1.

**TABLE 1:** New putative molecular targets, their underlying mechanisms involved with aging, and possible gero‐interventions.

New targets holding promise for therapeutic development	Evidence	Possible intervention (Observations)	Reference
RNA polymerase II (Pol II)	The speed of Pol II‐driven transcription increases with age, leading to more transcriptional errors in aged organisms (in *Drosophila, C. elegans*, mice and rats and in several human cell lines).	Slowing down Pol II elongation speed (?)	Debès et al.^32^
Imidazoline type 1 receptor (I1R)	The antihypertensive drug rilmenidine induces transcriptional changes similar to caloric restriction, improving the lifespan (in *C. elegans* and mice).	Agonists of I1R	Bennett et al.^41^
Nesprin‐2	Enhancing nuclear autophagy (‘nucleophagy’) delays aging (in *C. elegans* and mice).	Inducers/stabilizers of nesprins (?)	Papandreou et al.^36^
Protein phosphatase 2A (PP2A)	Aging brain is associated with a decline of PP2A activity (in zebrafish and mice).	Navitoclax (classic senolytic) or PP2A activators (i.e. MPH)	Xing et al.^18^
Acyl‐CoA binding protein (ACBP)	ACBP has a pro‐aging function (in yeasts, mice and humans).	Monoclonal anti‐ACBP antibodies (?)	Montégut et al.^11^
ZMPSTE24 (mammalian protease)	Treatment with certain protease inhibitors in patients living with HIV can cause premature aging.	Boosters of ZMPSTE24 (?)	Kuehnemann et al.^26^
FOXM1	FOXM1 expression declines during aging and an increase of its transcriptional activity delays organismal senescence and extends lifespan (in mice).	*[FOXM1 is also an oncogene so its induction to drive longevity must be considered with caution]*	Ouchi et al.^42^
GLS1 glutaminase, Glutamine metabolism	Glutamine metabolism sustains the viability of senescent cells and the biosynthesis of SASP.	Inhibitors of glutaminolysis (i.e. CB‐839, SP600125)	Johmura et al.^24^, Choudhury et al.^25^
Glycoprotein nonmetastatic melanoma protein B (GPNMB)	GPNMB is a seno‐antigen in senescent vascular endothelial cells.	Senolytic vaccine targeting the GPNMB	Suda et al.^22^
Bridge integrator 2 protein (BIN2)	Deletion of BIN2 in ovaries improves ovarian function and fertility in aged mice.	Cell‐penetrating peptides inhibitors of BIN2	Zhu et al.^38^
Vascular endothelial growth factor–A (VEGF‐A)	A moderate increase of VEGF‐A in the circulation leads to a notable lifespan extension (in mice).	Recombinant VEGF‐A (?)	Grunewald et al.^39^

There has been a lot of effort in identifying hallmark‐overarching markers that are predictive of age‐related morbidity and mortality, i.e. the so‐called biomarkers of healthy aging. These biomarkers could subsequently be used to identify vulnerable individuals in society before the development of age‐related diseases, so this can still be prevented, and to predict the outcome of interventions targeting aging. A perfect biomarker of healthy aging should (1) be inexpensive and minimally invasive to measure and show high reproducibility and accuracy, (2) predict age‐related disease and mortality more accurately than chronological age, and (3) ideally work equally well in humans and model organisms to increase translatability.^43^ Over the last couple of years, several blood‐based biomarkers have been identified in epidemiological studies based on different types of omics‐based measurements.^44^, ^45^ Much attention was given to the prediction of neurodegenerative diseases and a couple of interesting tracks are to be explored for the prediction of early cognitive impairment and dementia in older adults: the decline of the sense of smell,^46^ the level of toxic beta‐amyloid oligomers in the blood,^47^ or neuronal extracellular vesicles containing phosphorylated tau and alpha‐synuclein.^48^ From a broader perspective, the most promising biomarkers are those created based on clinically relevant outcomes, such as mortality, instead of chronological age.^49^, ^50^ As a next step, these biomarkers now need to be tested in more clinically relevant settings, such as in individuals visiting the hospital or general practitioners, to see if they (1) can indeed be used to identify vulnerable individuals on their way to develop age‐related diseases and (2) outperform or complement currently used health‐related markers for older adults in the clinic, such as the comprehensive geriatric assessment.

Our healthcare systems have been developing services capable of preventing adverse health outcomes for several life‐threatening events in aged populations, especially for complications of chronic diseases such as diabetes, hypertension, and obesity.^51^ Growing evidence shows that self‐management support interventions improve patient–level outcomes, especially when deployed in primary and community settings,^52^ and integrated with the modification of lifestyles.^53^ Despite the extension of the lifespan, the quality of life of older adults in terms of independent living and functionality is still far from being optimal, and this is largely caused by inadequate adherence to healthy lifestyles earlier in life, for which it is important to make changes at the individual level.^54^ Aging is a lifetime process, and lifestyle factors in midlife can predict successful aging 20 years later,^55^ thus providing a broad window of opportunity for interventions that can be personalized, especially when supported by innovative digital solutions.^56^ Indeed, lifestyles play a key role among non‐genetic factors affecting health and lifespan, especially food intake and activities stimulating physical and mental wellness, which deeply influence the molecular and physiological mechanisms underpinning major age‐related diseases.^57^ Pre‐symptomatic interventions and preventive care may be particularly effective in reducing frailty and extending a healthy lifespan.

By now, research and development in the biology of aging have moved to the mainstream with initiatives by large pharmaceutical companies and funds, highlighting the growing interest in geroscience and the development of interventional gerotherapeutics. For example, in 2023, Pfizer Ventures joined VitaDAO to fund longevity research projects and foster the spinouts of companies that conduct research in the area of longevity (https://www.vitadao.com/blog‐article/vitadao‐closes‐4‐1m‐fundraising‐round‐with‐pfizer‐and‐shine‐capital). Meanwhile, on the financing side, players like Altos and Hevolution have entered the field, looking to deploy massive amounts of capital to accelerate research and commercial product development (https://altoslabs.com/; https://hevolution.com). These initiatives reflect the increasing recognition of the potential commercial impact of anti‐aging therapies on healthcare and society.

Finally, why do we age? This question, which has so many facets — philosophical, spiritual, social, scientific, continues to trouble all those who hope one day we'll find the ‘fountain of youth’. Multiple theories of aging have been formulated based on individual aging effectors (i.e. genetic instability, ROS, mitochondrial damage, replicative senescence, etc.) but scientists are more and more convinced about the tight interconnection between the fundamental pathways of aging, undelaying a possible Unitary Theory of Fundamental Aging.^58^ This is raising the question of whether a therapeutic intervention on one mechanism would not negatively impact another one within the aging network. Experiments in worms and mice often demonstrated that one can make organisms live longer (by caloric restriction, for instance) but at the expense of some inherited dysfunctions or unhealthy lifespans.^59^, ^60^ In humans, centenarians and super‐centenarians may offer clues about the conditions necessary for slowing down the aging pace and living a long and healthy life. For instance, compared to old non‐centenarians, centenarians seem to display a unique, adapted peripheral immune profile (‘*immune resilience*’) that enables them to better recover from infections during their lives.^61^ However, it is not clear whether this adaptation of the immune system is the cause or the effect of the slow aging in centenarians. A ‘*transcriptome imbalance* ’ has been recently described as a cause of aging in both mice and humans.^62^ This is based on the abundance of short RNA transcripts over the long transcripts in many aged tissues, which implies a shift towards the transcription of small genes during aging; this process can be reversed by several gero‐therapeutics in mice but a causal relationship with the transcription shift was not demonstrated yet.

Thus far, there is no evidence that human aging at the organismal level can be stopped and reversed, as was shown in lower organisms and laboratory animals. Based on the documented epigenetic modifications during aging^63^ (i.e. DNA methylation, post‐translational modifications of histones, heterochromatin alteration), that most likely drive over the genetic determinants, cellular reprogramming with Yamanaka factors and epigenetic resetting ^64^, ^65^ are the most promising possibilities to reverse aging in mammals. These approaches could reverse the biological age, at least in certain organs/tissues, if not at the systemic level.^66^

Another still debated problem around the rejuvenation strategies is how well currently used biomarkers of healthy aging can actually quantify aging and rejuvenation. So far, the epigenetic clocks based on DNA methylation at specific CpG sites have proven to be the most promising in estimating both the health state of organs and tissues^67^ and the mammalian biological age after longevity interventions.^68^

Ultimately, Covid‐19 and its systemic complications (especially those of ‘long Covid’) can induce modifications in the onset and evolution of several chronic diseases in aged individuals^69^, ^70^ and also on epigenetic aging (as assessed by DNA methylation).^71^ Therefore, efforts to study the impact of SARS‐CoV‐2 infection (and also of other related coronaviruses) on the mechanisms of aging are to be considered in future studies.

Identifying new pathways (‘pillars’) of such a complex process like aging and druggable targets as possible therapeutic interventions, will remain a very hot area for personalized medicine and human well‐being. However, most likely, these interventions will need to be applied together with personalized and digitally supported measures to enhance lifestyles, like healthy eating and physical exercise, to open the way, if ever, to “the fountain of youth”.

## AUTHOR CONTRIBUTIONS

**Iuliana POPESCU:** Conceptualization (lead); project administration (lead); supervision (lead); visualization (lead); writing – original draft (lead); writing – review and editing (lead). **Joris Deelen:** Writing – review and editing (equal). **Maddalena Illario:** Writing – review and editing (equal). **Jan Adams:** Writing – review and editing (equal).

## FUNDING INFORMATION

This work was not supported by any particular fund.

## CONFLICT OF INTEREST STATEMENT

I.P., J.D. and M.I. declare no conflict of interest. J.A. is a Partner at Apollo Health Ventures, which has a financinal interest in Samsara Therapeutics.

## Data Availability

Data sharing not applicable to this article as no datasets were generated or analysed during the current study.
